# How sensitive are predicted muscle and knee contact forces to normalization factors and polynomial order in the muscle recruitment criterion formulation?

**DOI:** 10.1080/23335432.2018.1514278

**Published:** 2018-09-10

**Authors:** Michael Skipper Andersen

**Affiliations:** Department of Materials and Production, Aalborg University, Aalborg, Denmark

**Keywords:** Musculoskeletal modeling, inverse dynamics, force-dependent kinematics, muscle recruitment, knee contact forces, sensitivity

## Abstract

Musculoskeletal modeling is an important tool to estimate knee loads. In these models, anatomical muscles are frequently sub-divided to account for wide origin/insertion areas. The specific sub-division has been shown to affect some muscle recruitment criteria and it has been suggested that normalization factors should be incorporated into models. The primary aim of this study was to investigate the effect of different muscle normalization factors in the muscle recruitment criterion and polynomial order on the estimated muscle and total, medial and lateral knee contact forces during gait. These were evaluated on three different musculoskeletal models with increasing levels of patient-specificity and knee joint model complexity for one subject from the Grand Challenge data set and evaluated against measured forces. The results showed that the introduction of the muscle normalization factors affected the estimated forces and that this effect was most pronounced when a polynomial of order two was applied. Additionally, mainly the second contact force peak was affected. Secondary investigations revealed that the predicted forces can vary substantially as a function of the knee flexor and extensor muscle strength with over one body weight difference in predicted total compressive force between 100% and 40% of the strength. Additionally, the predicted second peak during gait was found to be sensitive to the position of the pelvic skin marker positions in the model. These results imply that caution should be taken when a normalization factor is introduced to account for sub-divided muscles especially for second-order recruitment criteria.

## Introduction

Within multiple areas, such as clinical gait analysis (Zajac et al. 2002) and orthopedics (Mellon et al. 2013; Marra et al. 2015), knowledge of muscle, ligament and contact forces are important. However, due to the inability to measure muscle, ligament and contact forces non-invasively, musculoskeletal models are often applied to estimate these and several solution approaches are available in the literature ranging from Electromyography (EMG)-driven models (Buchanan et al. 2004), forward dynamics-based tracking methods (Thelen and Anderson 2006) and inverse dynamics-based models (Crowninshield 1978; Rasmussen et al. 2001). Additionally, rather than applying idealized joint models, advanced simulation frameworks have been developed to enable analysis of models with detailed joint descriptions, such as the enhanced Computed Muscle Control (CMC) algorithm (Thelen et al. 2014) and Force-dependent Kinematics (FDK) (Andersen et al., 2017).

With muscle forces being the largest contributor to joint contact forces (Herzog et al. 2003), it is important for the accuracy of the predictions how the muscles are modeled. In forward dynamics-based tracking and inverse dynamics-based simulations (Crowninshield 1978; Rasmussen et al. 2001), assumptions about how the muscles are recruited are necessary to resolve the muscle redundancy problem (Erdemir et al. 2007). This is typically accomplished by introducing an optimality criterion, frequently expressed as minimization of the sum of muscle activities (defined as muscle force divided by muscle strength) to some power (Praagman et al. 2006), the maximum muscle activity (Rasmussen et al. 2001), energy (Praagman et al. 2006) or a weighted least-square (Knarr and Higginson 2015).

Recent literature has demonstrated both theoretically and with numerical examples that the polynomial muscle recruitment criterion is sensitive to the sub-division of the anatomical muscles into smaller muscle-tendon units to capture large origin/insertion regions (Holmberg and Klarbring 2011). As described by Holmberg and Klarbring (2011), the polynomial recruitment criterion will produce different estimated muscle forces if a muscle is discretized into multiple units even if they all have the same origin and insertion path as the original muscle. This is an undesirable feature of this type of muscle recruitment as this effect has not been taken into account when datasets, such as the Twente Lower Extremity Model (TLEM) 1.0 (Horsman et al. 2007) and TLEM 2.0 (Carbone et al. 2015) were created and some muscles were split up into multiple branches. It is, therefore, desirable to build a correction of this behavior into the muscle recruitment criterion. To this end, Holmberg and Klarbring (2011) proposed a correction method for the polynomial recruitment criterion and also noted that the min-max criterion (Rasmussen et al. 2001) is not sensitive to sub-divided muscles. The idea of the correction method of Holmberg and Klarbring (2011) is to ensure that, when a muscle is subdivided and all subdivisions have the same origins and insertions, the total force production of the subdivisions is the same as the original muscle. Although not proposed as a method to correct for sub-divided muscles, Happee and Van der Helm (1995) proposed a volume-weighted muscle recruitment criterion, and as sub-divided muscles are assigned a fraction of the total muscle volume, this criterion has a built-in correction for the sub-division of the muscles. The effects of including these types of correction factors into models estimating the medial and lateral knee contact forces has not been studied before.

Another important aspect of the musculoskeletal model is how the model geometry, muscle-tendon parameters and kinematics are described. There have been some recent studies that have analyzed the effects of including subject-specific geometric information as compared to scaled generic models. Gerus et al. (2013) investigated the effect of including subject-specific geometry and/or knee kinematics in an EMG-driven model and found the most accurate predictions for a subject-specific model that utilized minimization of the peak tibiofemoral contact force in a calibration process. Scheys et al. (2008) studied the estimated moment arms and muscle-tendon lengths between scaled generic and MRI-based models and found all tested generic models failed to accurately estimate these parameters. As the predictions of muscle forces of musculoskeletal models are sensitive to the musculoskeletal geometry (Carbone et al. 2012), it is expected that the predicted tibiofemoral joint contact forces will be affected by these differences, although this, as far as we know, has not been specifically evaluated yet in inverse dynamics-based simulations tailored to predict knee contact forces.

With the development of instrumented implants, there has been increased attention in the modeling community on direct model validation, e.g. through the Grand Challenge Competition to Predict *In Vivo* Knee Loads (Fregly et al. 2012b). However, while this presents an excellent opportunity for model validation, the osteoarthritis population is not healthy. For instance, reduced isometric knee flexion and extension strength following Total Knee Arthroplasty (TKA) has been reported to be 31% on average but with up to 40% at low knee flexion angles (Silva et al. 2003). Since the said muscle recruitment criteria typically dependent on muscle strength estimates, it is unknown how this reduction in knee flexor and extensor strength affects the model outputs and how it interacts with the muscle recruitment formulation. Additionally, the TKA patients are frequently overweight and obesity is a widely acknowledged risk factor for the development osteoarthritis (Bliddal et al. 2014). Therefore, when performing kinematic analysis of these patients using skin marker-based motion capture, it may be challenging to place the markers on the model in the correct position due to the amount of soft tissue between the skin and the underlying bony landmarks. This is particularly challenging in the abdomen region, where the pelvic markers are located.

Therefore, the main purpose of this study was to investigate how different muscle recruitment criterion formulations with and without normalization factors to correct for sub-divided muscles affect the predictions of the total, medial and lateral knee contact forces during gait. Two secondary aims were to investigate how sensitive the contact force predictions are to the modeled isometric strengths of the knee flexors and extensors, and the modeled positions of pelvic skin markers. To keep the number of figures and tables manageable, these secondary aims were only investigated in a patient-specific model with a revolute knee joint.

## Methods

### Experimental data

In this study, we used the standing reference trial and gait data from the 5th Grand Challenge Competition to Predict *In Vivo* Knee Loads (Fregly et al. 2012b). This data were collected from one male subject (age: 86, height: 1.80 m and mass: 75 kg) with an instrumented, posterior cruciate-retaining TKA prosthesis, measuring the knee forces and moments. Among others, the dataset contain measurements from the knee prosthesis (denoted eTibia), trajectories of skin markers and ground reaction forces measured during standing reference trials and various activities of daily living, including gait at a self-selected speed and pre- and post-operative Computed Tomography (CT) scans. The data set also include Stereolithography (STL) 3D geometries of the femoral component, tibial tray and insert, patellar button, and segmentations of the post-operative CTs of the partial pelvis, femur, patella, tibia, fibula, partial talus and partial calcaneus.

In this study, we apply the CT data, the STL files of the bone and prosthesis components and the movement data from the standing reference trial (PS_staticfor2) and the one gait trial at self-selected speed (PS_ngait_og_ss1) that was part of the 2014 competition and utilized in the model of Marra et al. (2015). The CT data were applied to create patient-specific bone geometries and morph the muscle attachment sites in a patient-specific model. The STL files were used to create a detailed knee model (see the section ‘Patient-specific models’ below for details).

### Models

Three different musculoskeletal models were built and analyzed using the AnyBody Modeling System v. 6.1 (AnyBody Technology A/S, Denmark) and the AnyBody Managed Repository (AMMR) v. 1.6 but with the model data for the lower extremity updated to the TLEM 2.0 data set. The models, with increasing patient-specificity and knee joint model complexity, were:

(1) A linearly scaled model with a hinge knee joint derived based on the linearly scaled model of Lund et al. (2015).
(2) A patient-specific model with a hinge knee joint from Marra et al. (2015).
(3) A patient-specific model with an 11-DOF FDK-based knee joint from Marra et al. (2015).

#### Patient-specific models

The patient-specific models were identical to the models developed and validated by Marra et al. and are described in detail in Marra et al. (2015). Briefly, the cadaver-based geometry of the TLEM 2.0 data set was nonlinearly morphed to the pre-operative patient-specific geometry based on bones segmented from CT. For the CT bones, the hip joint center, and the tibiofemoral, patellofemoral and the talocrural joint centers and axes were identified using sphere and cylinder fitting, respectively. After morphing, the post-operative bones and implant components were registered onto the morphed musculoskeletal model. As the CT scans did not include the full pelvis, talus and feet, the skin markers from the standing reference trial were used to scale the size of these using the algorithm of Andersen et al. (2010). This approach required identification of the location of the skin markers on bony landmarks on the TLEM 2.0 geometry, which introduces a degree of uncertainly especially for the pelvis markers, as we shall investigate with a sensitivity study.

After the model scaling was completed, the markers during the gait trial were tracked by minimizing the least-squares difference between modeled and experimental markers (Andersen et al. 2009). In this process, a hinge knee was applied. Subsequently, two different inverse dynamics-based simulations were performed: (1) with a hinge knee joint model and (2) with an 11-Degree of Freedom (DOF) FDK-based knee joint model. The FDK-based model included an elastic foundation model for the knee contact surfaces, as well as nonlinear ligament models and resolved the muscle, ligament and contact forces and secondary knee joint kinematics by utilizing a quasi-static force equilibrium approach for each time step. As part of this process, a muscle recruitment criterion is required, and several different objective functions were evaluated as shall be explained later. Details about the specific contact models and ligament properties are described in Marra et al. (2015) and the theory behind FDK is described in Andersen et al. (2017). For the FDK-based knee model, the total, medial and lateral contact forces were derived directly from the estimated forces on each contact surface. However, when the hinge knee model was applied, medial and lateral contact forces were estimated using regression equations, based on the net force and moment at the location of the eTibia transducer, provided in the Grand Challenge data set.

#### Linearly scaled model

The linearly scaled model (Lund et al. 2015) was developed using the same cadaver-based model as the patient-specific models, but with a different approach to model scaling. Otherwise, the same marker tracking and inverse dynamic analysis approach during the gait trial as the patient-specific model with the hinge knee joint was applied. For scaling, the standing reference trial was applied. First, the markers located on anatomical landmarks and the feet were manually placed on the model. Secondly, the optimization problem of Andersen et al. (2010) was solved to scale the dimensions of the fore- and upper-arms, thorax, pelvis, thigh, shank, and feet to minimize the least-squares difference between modeled and experimental markers. Finally, the location of all cluster markers were computed in the segment coordinate systems and saved together with the estimated segment lengths. For the inverse kinematics and inverse dynamic analysis, standard approaches were applied, where the inverse kinematics minimized the least-squares difference between modeled and experimental markers (Andersen et al. 2009) and inverse dynamics was performed by solving the muscle recruitment problem (see below). To distribute the total contact forces and frontal moment to an estimate of medial and lateral contact force, an equilibrium in the frontal plane was setup and the moment arms of the medial and lateral contact force estimated based on (Seedhom et al. 1972) with the knee width estimated based on the skin markers.

### Muscle model and strength scaling

Each muscle unit was modeled using a Hill-type muscle model (Zajac 1989) based on the TLEM 2.0 dataset (Carbone et al. 2015) and was used to estimate the instantaneous muscle strength,$\;s_{i}$, based on muscle length and contraction velocity. The instantaneous muscle strength is defined as the maximal force that the muscle contractile element can produce given its current length and velocity. The specific force–length and force–velocity relationships applied in the AnyBody Modeling System are adopted from Daxner (1997) and Gföhler et al. (1999).

The nominal isometric strength was estimated based on the muscle Physiological Cross Sectional Area (PSCA) multiplied by 27 N/cm^2^ as derived from cadaver studies by Horsman et al. (2007) and scaled using a length–mass scaling law as proposed by Rasmussen et al. (2005). Additionally, the isometric strength of the knee flexions and extensors were reduced by 35% as in Marra et al. (2015) to mimic the reported strength reduction of TKA patients (Silva et al. 2003). Additionally, to account for model scaling, the AMMR has built-in calibration procedures for the tendon slack lengths that are always executed when the Hill-type muscle model is applied. In this study, we applied the one-step calibration, which, after the leg is scaled, places the leg in series of poses, each representing the situation in which the individual muscles are assumed to have its tendon exactly slack. The muscle parameters are then updated with these tendon slack lengths for all subsequent inverse dynamic analysis.

### Muscle recruitment problem

Due to the number of muscles compared to the number of DOF in the musculoskeletal model, infinitely many combinations of muscle forces can theoretically fulfill the dynamic equilibrium equations of motion.

The muscle recruitment problem was formulated as a polynomial optimization problem, minimizing a scalar objective function, *G*, subject to the dynamic equilibrium equations and inequality constraints, specifying that muscles cannot push, only pull. As described by Holmberg and Klarbring (2011), sub-division of muscles can affect the estimated muscle and joint reaction forces unless accounted for in the objective function. Therefore, several suggestions for how this compensation can be achieved are available in the literature (Happee and Van Der Helm 1995;Holmberg and Klarbring 2011). Common to these is the introduction of a normalization factor for each muscle that we denote $n_{i}$ for the *i*th muscle:

$$\underset{f}{\min}G\left(f\right)=\;\overset{n^{\left(M\right)}}{\underset{i=1}{\sum }}n_{i}{\left(\frac{f_{i}^{\left(M\right)}}{s_{i}}\right)}^{p}$$

(1) Cf=d

$$0\leq f_{i}^{\left(M\right)}\leq s_{i},\;i=1,\;\ldots ,\;n^{\left(M\right)}.$$

$f_{i}^{\left(M\right)}$ is the ith muscle force, $n^{\left(M\right)}$ is the number of muscles and $s_{i}$ is the strength of the muscle. **C** is the coefficient matrix for the unknown muscle and joint reaction forces, f, and **d** contains all external loads and inertia forces. p is the polynomial order.

### Sensitivity studies

To investigate the effect of the muscle recruitment formulation on the muscle and joint contact forces, all models were solved in nine different setups given by the combination of the muscle normalization factor and the polynomial order. For the normalization factors, we analyzed:

1. No normalization given by $n_{i}=$1.

(2) Normalization based on the muscle volume, where the total muscle volume was evenly distributed between sub-divided muscle branches, i.e. $n_{i}=V_{i}/n_{i}^{s}$ . Where $V_{i}$ is the volume of the anatomical muscle and $n_{i}^{s}$ is the number of elements in the sub-divided anatomical muscle that the *i*th muscle is a part of. An example of this is the soleus medialis in the TLEM 2.0 dataset, which is sub-divided into three elements. Hence, for these three muscles in the muscle recruitment, $n_{i}^{s}=3$ and $V_{i}$ is the same for all three.

(3) Normalization based on the number of sub-divided muscles as proposed by Holmberg and Klarbring (2011). Note, however, that Holmberg and Klarbring (2011) derived the correction factor as an adjustment of the muscle strength but this we rewrote into an equivalent normalization factor for Equation (1), i.e. $n_{i}=1/n_{i}^{s}$. For each of these three normalization factors, we analyzed with the polynomial order *p* set to 2, 3 and 4.

**Figure 1.**
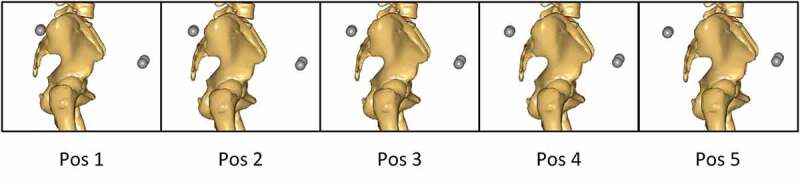
Illustration of the five different anterior–posterior positions of the ASIS and PSIS markers evaluated. From left to right, the markers are moved 1 cm posteriorly in the pelvic anatomical frame from position to position

Subsequently, two additional investigations were performed only with the patient-specific model with a hinge knee:

(4) The strength of the knee flexors and extensors were reduced from 100% to 40% in steps of 10% of the baseline strength estimated by the length–mass–fat scaling law.

(5) The assumed position of the four (left and right Anterior Superior Iliac Spine (ASIS) and left and right Posterior Superior Iliac Spine (PSIS)) pelvis markers on the model were systematically moved in the anterior-posterior direction by positioning them at five different locations as illustrated in Figure 1. Note that the anterior direction is defined based on the anatomical reference frame of the pelvis and is, therefore, tilted slightly downward as specified by the ISB recommendations (Wu et al. 2002). The distance from the PSIS to the ASIS markers was kept fixed and estimated based on the skin markers in the standing reference trial.

### Data analysis

For each model and each simulation setup, we extracted the muscle tendon forces for selected muscles important for knee biomechanics, total, medial and lateral contact force estimates and resampled these to 100% of the gait cycle at an interval of 1% and normalized the amplitudes to body weight. The measured total, medial and lateral contact forces from the eTibia were also extracted, resampled to 100% of the gait cycle and normalized to body weight. For the analysis that we completed for all three models, we quantified the Root-Mean-Square-Difference (RMSD) and squared Pearson’s correlation coefficient, *r*^2^, between estimated and measured total, medial and lateral contact force. As some muscle in the TLEM data set are divided into multiple branches, for each muscle, we extracted the force envelope of the individual branches.

The squared Pearson’s correlation coefficient was quantified as ‘weak’ (*r*^2^ ≤ 0.35), ‘moderate’ (0. 35 < *r*^2^ ≤ 0.67), ‘strong’ (0.67 < *r*^2^ ≤ 0.90) and excellent (*r*^2^ > 0.90) (Taylor 1990).

## Results

**Figure 2.**
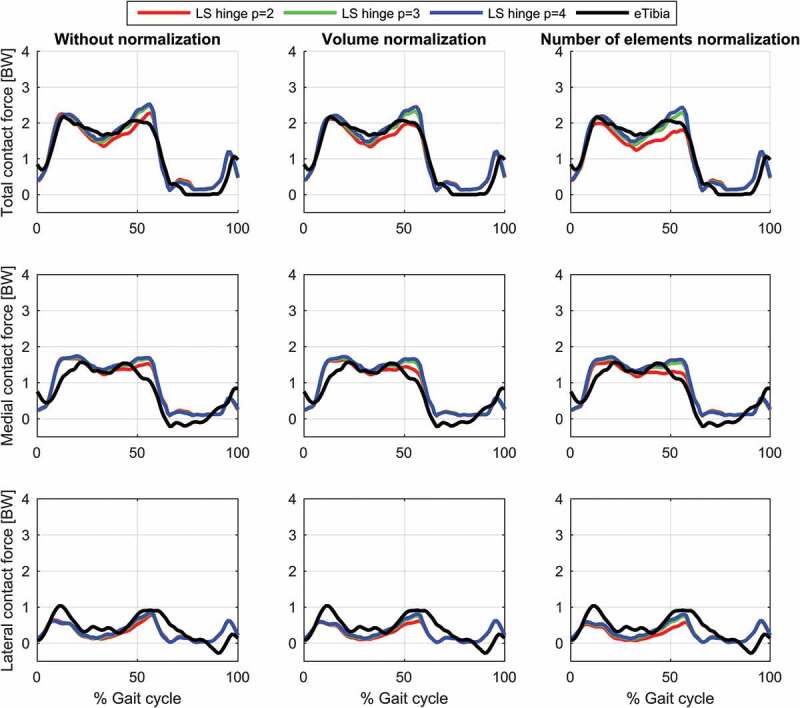
Contact force results for the linearly scaled model with a hinge knee joint during a gait cycle. Estimations of the total (top), medial (middle) and lateral (bottom) contact forces during a gait cycle plotted together with the measured forces for the different muscle recruitment formulations. LS: Linear scaling

**Figure 3.**
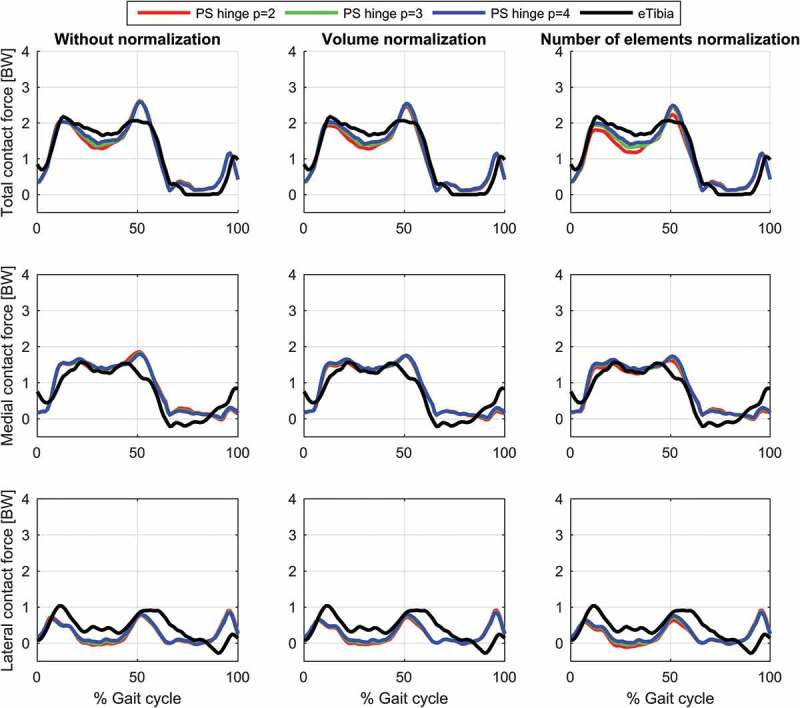
Contact force results for the patient-specific model with a hinge knee joint during a gait cycle. Estimations of the total (top), medial (middle) and lateral (bottom) contact forces during a gait cycle plotted together with the measured forces for the different muscle recruitment formulations. PS: Patient-specific scaling

**Figure 4.**
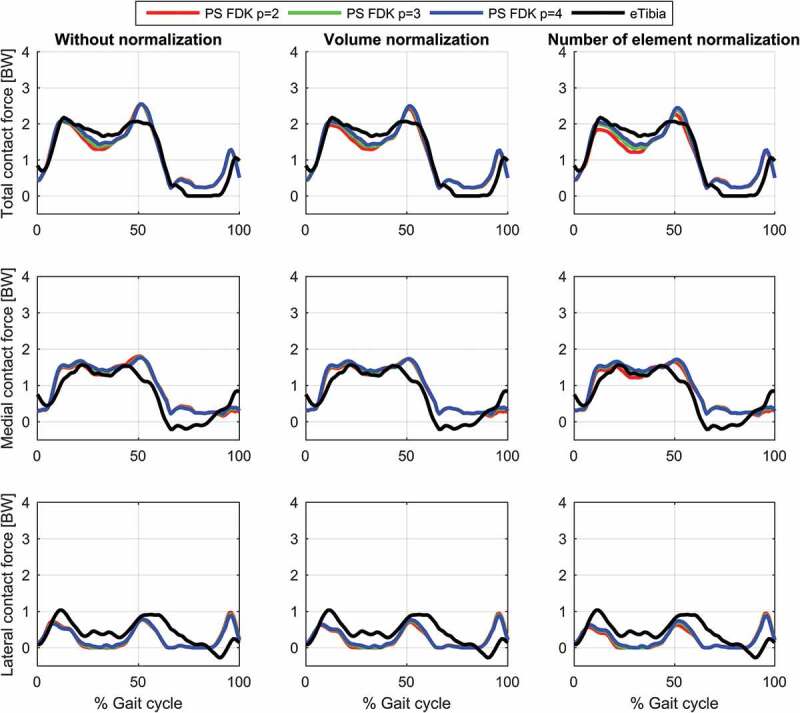
Contact force results for the patient-specific model with an 11-DOF FDK-based knee joint during a gait cycle. Estimations of the total (top), medial (middle) and lateral (bottom) contact forces during a gait cycle plotted together with the measured forces for the different muscle recruitment formulations. PS: Patient-specific scaling

The results for the study of the normalization factors and polynomial order of the muscle recruitment problem for the different models on estimated contact forces are depicted in Figures 2–4 and the corresponding RMSD and squared Pearson’s correlation coefficients are presented in Tables 1 and 2, respectively.

**Table 1.** RMSE (in body weight) of the estimated total (top), medial (middle) and lateral (bottom) contact force compared to the measured forces for different combinations of polynomial order (2, 3 and 4) and muscle normalization approach (without, volume and number of elements) for each of the three models

	Without normalization			Volume normalization			No. elements normalization		
	*p* = 2	*p* = 3	*p* = 4	*p* = 2	*p* = 3	*p* = 4	*p* = 2	*p* = 3	*p* = 4
**Total contact force**									
PS FDK	0.30	0.28	0.27	0.28	0.27	0.26	0.32	0.28	0.26
PS hinge	0.29	0.26	0.25	0.28	0.26	0.24	0.32	0.27	0.25
LS hinge	0.24	0.24	0.24	0.23	0.22	0.23	0.28	0.22	0.22
**Medial contact force**									
PS FDK	0.34	0.33	0.33	0.31	0.32	0.32	0.31	0.31	0.32
PS hinge	0.33	0.31	0.31	0.29	0.30	0.30	0.28	0.29	0.29
LS hinge	0.29	0.30	0.31	0.26	0.28	0.30	0.24	0.28	0.30
**Lateral contact force**									
PS FDK	0.37	0.36	0.34	0.38	0.36	0.34	0.39	0.37	0.35
PS hinge	0.38	0.36	0.34	0.38	0.36	0.34	0.41	0.37	0.35
LS hinge	0.27	0.27	0.27	0.26	0.28	0.27	0.32	0.28	0.27

**Table 2.** Squared Pearson’s correlation coefficient, *r*^2^, of the estimated total (top), medial (middle) and lateral (bottom) contact force compared to the measured forces for different combinations of polynomial order (2, 3 and 4) and muscle normalization approach (without, volume and number of elements) for each of the three models

	Without normalization			Volume normalization			No. elements normalization		
	*p* = 2	*p* = 3	*p* = 4	*p* = 2	*p* = 3	*p* = 4	*p* = 2	*p* = 3	*p* = 4
**Total contact force**									
PS FDK	0.86	0.88	0.90	0.89	0.89	0.90	0.88	0.89	0.90
PS hinge	0.87	0.89	0.90	0.89	0.90	0.91	0.89	0.90	0.91
LS hinge	0.91	0.92	0.93	0.93	0.93	0.93	0.92	0.93	0.93
**Medial contact force**									
PS FDK	0.78	0.81	0.82	0.80	0.81	0.82	0.79	0.81	0.82
PS hinge	0.78	0.81	0.82	0.81	0.82	0.83	0.81	0.82	0.82
LS hinge	0.82	0.84	0.85	0.85	0.85	0.85	0.85	0.85	0.85
**Lateral contact force**									
PS FDK	0.18	0.21	0.24	0.15	0.20	0.23	0.11	0.18	0.21
PS hinge	0.15	0.18	0.20	0.12	0.17	0.20	0.08	0.14	0.18
LS hinge	0.43	0.43	0.43	0.38	0.41	0.42	0.31	0.40	0.41

For the same combination of normalization factor and polynomial order, among the evaluated models, the linearly scaled model demonstrated the lowest RMSDs and highest correlation coefficients although the differences were minor for the total and the medial contact force. The correlation coefficients were bordering between strong and excellent correlations for the total contact force and the medial contact force remained in the strong correlation interval. In the lateral contact force, however, both patient-specific models demonstrated a weak correlation in all cases, whereas the linearly scaled model showed a ‘moderate’ correlation.

The results showed a nonlinear effect of the normalization factor and an interaction with the polynomial order for the contact forces. In all cases, normalizing by the number of muscle elements or the volume lowered the estimated contact forces compared to no normalization and with the effect being more pronounced with a polynomial power of two and minor for four. Of the two normalization factors, normalizing by the number of elements had the largest impact on the predictions especially around the second peak.

**Figure 5.**
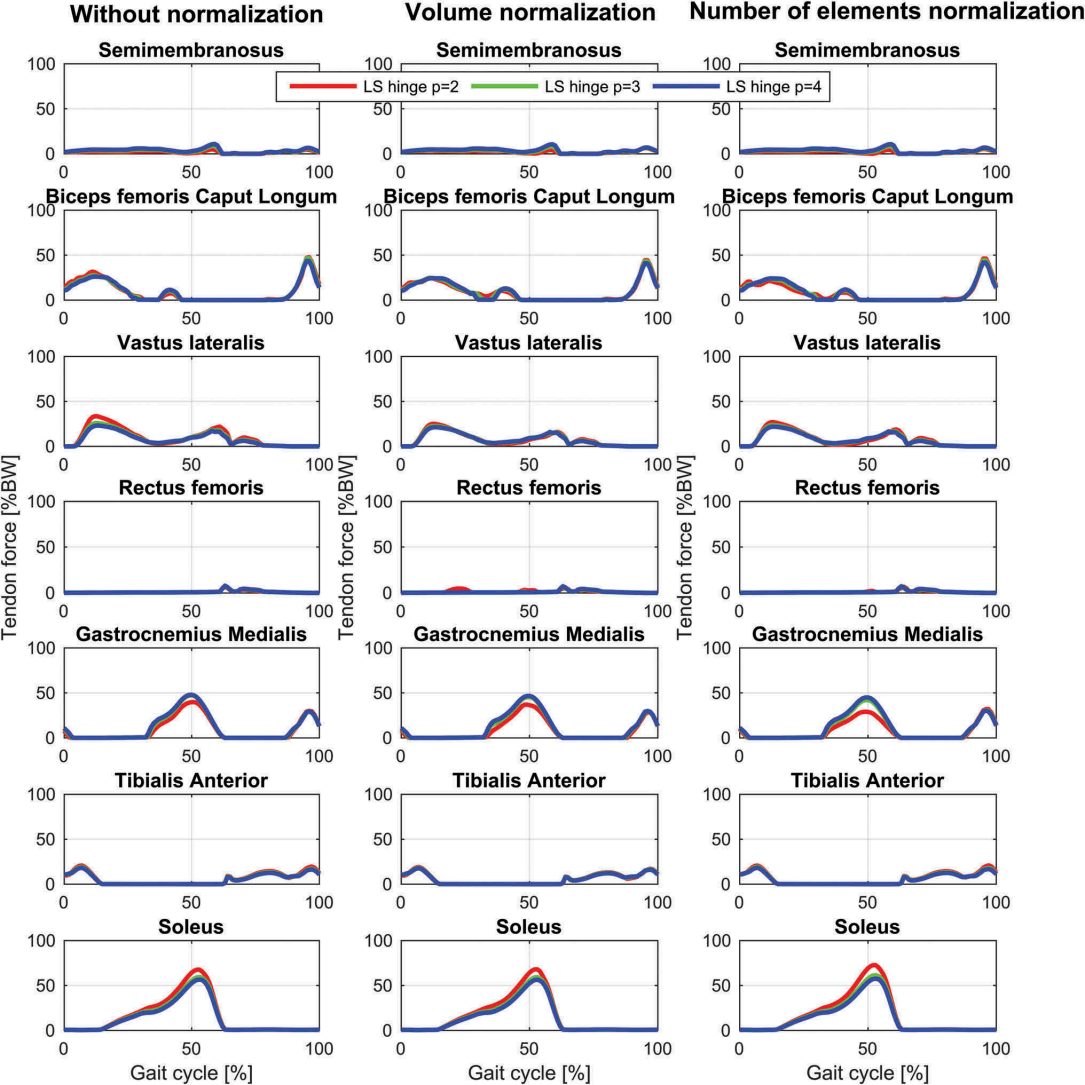
Muscle force results for selected muscles with the linearly scaled model with a hinge knee joint during a gait cycle for the different muscle recruitment formulations. LS: Linear scaling

**Figure 6.**
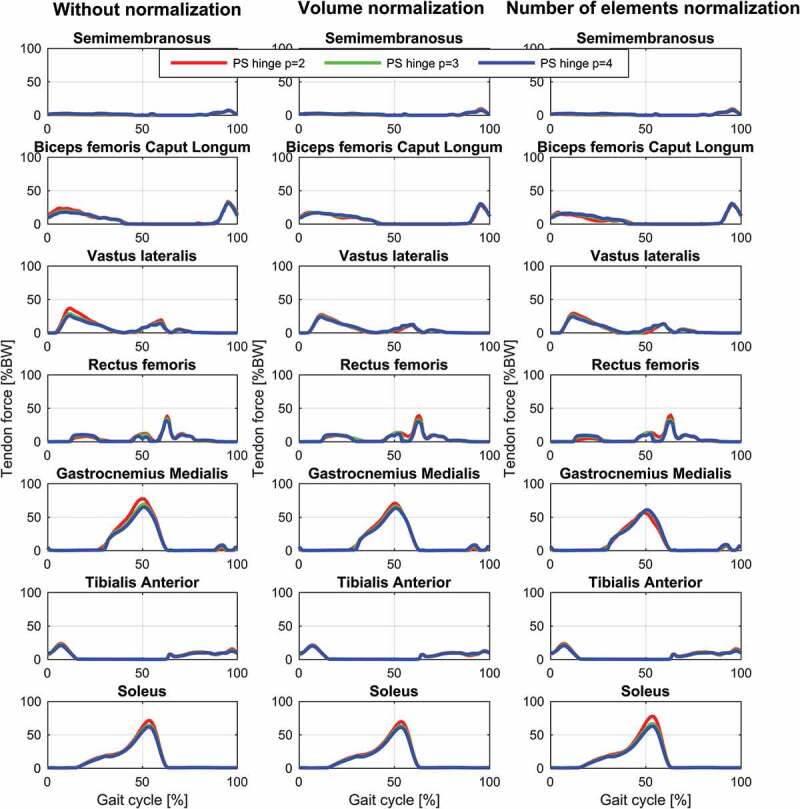
Muscle force results for selected muscles with the patient-specific model with a hinge knee joint during a gait cycle for the different muscle recruitment formulations. PS: Patient-specific scaling

**Figure 7.**
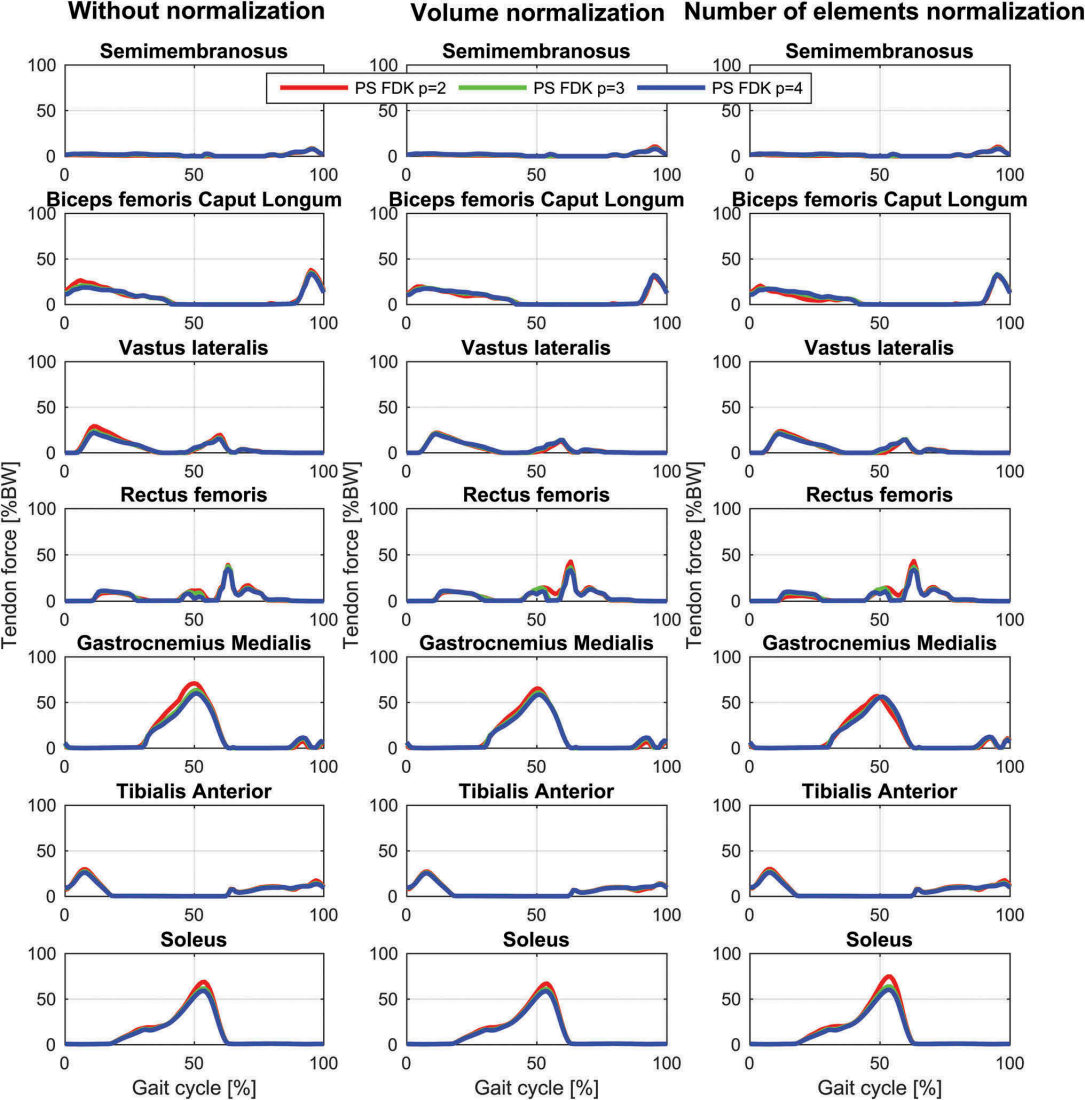
Muscle force results for selected muscles with the patient-specific model with an 11-DOF FDK-based knee joint during a gait cycle for the different muscle recruitment formulations. PS: Patient-specific scaling

Estimated muscle forces for the different muscle recruitment criteria and models are depicted in Figures 5–7. Generally, the recruitment patterns and timing are the same for all models and criteria but with small differences. The general trend in the models is that the muscle forces are decreasing when the polynomial order is increased except for gastrocnemius with the linearly scaled model that shows the opposite trend and minute changes for biceps femoris caput longum with the number of elements normalization that demonstrate a slight increase with increased power during mid-stance. Additionally, the linearly scaled model shows a generally lower gastrocnemius force and rectus femoris force during early swing.

**Figure 8.**
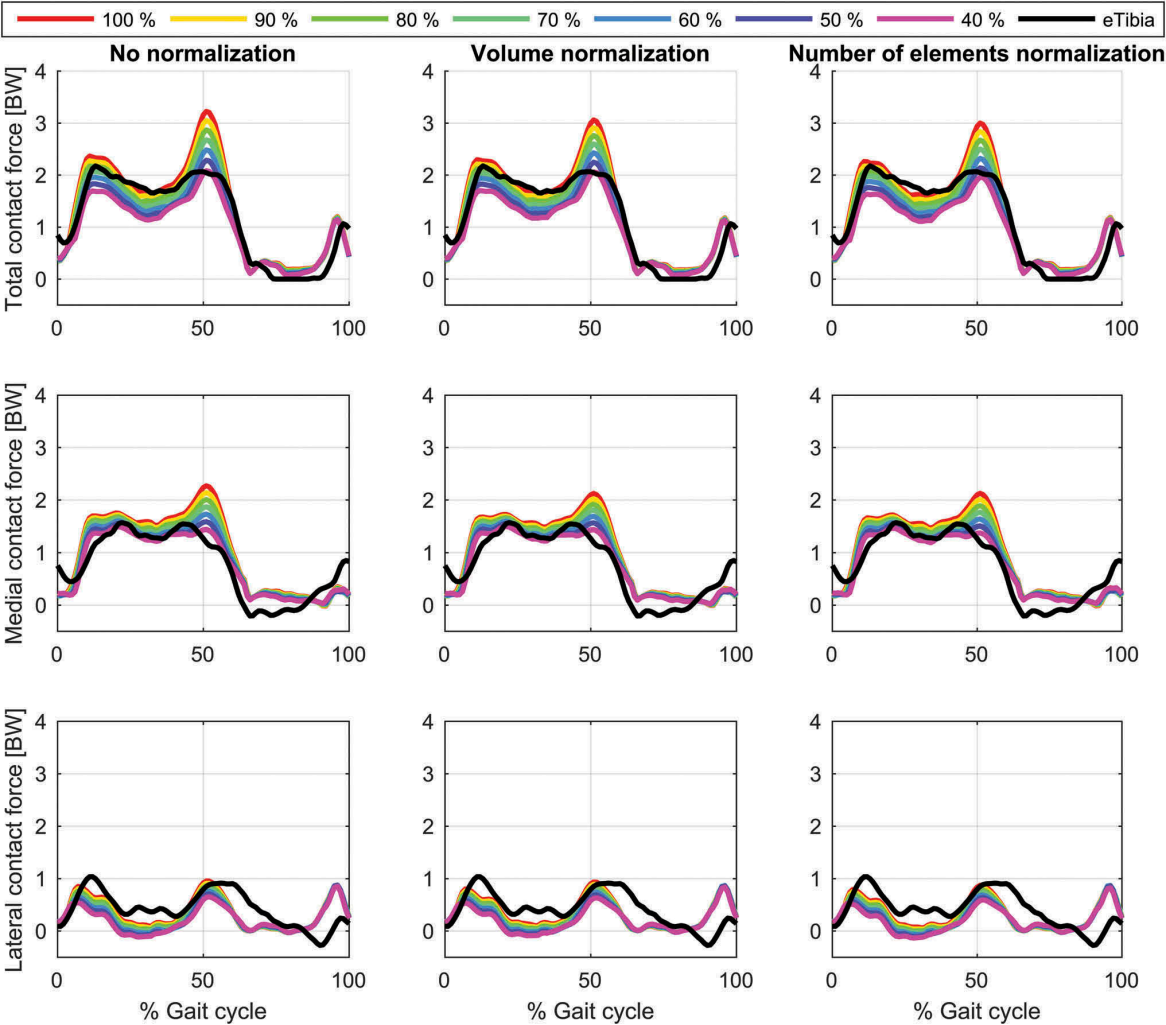
Variations in knee contact forces as a function of isometric knee flexor and extensor strength evaluated for the patient-specific model with hinge knee joint and a polynomial order of three in the muscle recruitment criterion. The figure depicts the results for the total (top), medial (middle) and lateral (bottom) contact force without muscle normalization (first column), with volume normalization (second column) and with normalization based on number of elements (third column). The actual muscle strength is given as a percentages of the strength of the muscles estimated by the length-mass-fat scaling law, e.g. 40% means that the strength has been reduced to 40%

The predicted contact forces showed a strong sensitivity to the isometric strength of the knee flexors and extensors with an increase in the predicted knee contact forces during the stance phase when the isometric strength was increased (Figure 8). Both the first and the second peak of the estimated force were affected but with a larger effect on the second peak for the total and medial contact forces.

**Figure 9.**
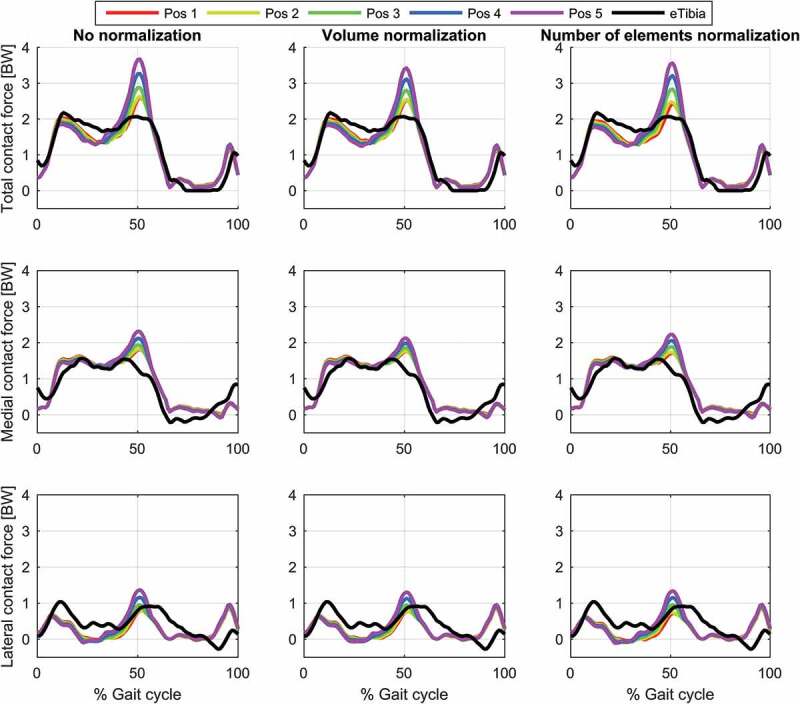
Variations in knee contact forces as a function of anterior-posterior pelvis marker positions evaluated for the patient-specific model with hinge knee joint and a polynomial order of three in the muscle recruitment criterion. The figure depicts the results for the total (top), medial (middle) and lateral (bottom) contact force without muscle normalization (first column), with volume normalization (second column) and with normalization based on number of elements (third column)

Finally, the anterior–posterior position of the pelvis markers had a large impact on the estimation of the second contact force peak with the lowest estimated second peak of the total contact force being around 2.5 BW to around 3.5 BW between positions 1 and 5, respectively (Figure 9). The introduction of muscle normalization factors had no marked effect on this sensitivity.

## Discussion

In this study, we investigated the effects of different muscle recruitment criteria formulations on the predictions of the muscle forces and total, medial and lateral knee contact forces. This was performed on three models: (1) a linearly scaled cadaver-based model with a hinge knee joint, (2) a model with patient-specific geometry but still a hinge knee and (3) a model with patient-specific geometry and an 11-DOF FDK-based knee joint. Additionally, on the patient-specific model with a hinge knee joint, we investigated the sensitivity of the predictions to the modeled isometric strength of the knee flexors and extensors and, finally, the sensitivity to the placement of the pelvis markers in the anterior–posterior direction were evaluated.

There is a current trend in musculoskeletal modeling toward patient-specificity and higher and higher anatomical accuracy and, as part of this, high fidelity representations of the geometry of the muscles. Two examples of this are the TLEM (Horsman et al. 2007; Carbone et al. 2015) and the Twente spine (Bayoglu et al. 2017) data sets which both included sub-divided muscles to better capture the origin and insertion areas as compared to single element representations. For this reason, it is important that the analysis approaches applied using these data do not include undesirable effects, such as different predictions of muscle forces due to sub-divided muscles even if the different branches have the same origin and insertion path as the not divided muscle.

The investigation of the muscle recruitment criterion, including both the normalization factor, as well as the polynomial order, showed an effect primarily on the second peak contact force and the effect was largest with the second-order polynomial criterion. The largest forces were found with no normalization, second largest with the volume normalization and finally the normalization based on the number of elements showed the lowest forces. As explained by Rasmussen et al. (2001), the polynomial recruitment criterion approaches the min–max criterion for the polynomial order approaching infinity and, since Holmberg and Klarbring (2011) showed that the min-max criterion is unaffected by the muscle discretization, it is logical that the most affected criterion is the second-order criterion and the least affected is the fourth-order criterion. Of the three different normalization factors in the muscle recruitment criterion formulations, volume normalization appears to have the clearest physiological interpretation. In this case, ${\left(\frac{f_{i}^{\left(M\right)}}{s_{i}}\right)}^{p}$in Equation (1) represents the cost of activating a unit volume of the specific muscle element. With multiplication of each unit cost by the muscle element volume summed over all muscle volumes, this criterion minimizes the cost of the entire muscle volume. While the specific cost of activating a unit volume of a muscle remains to be determined, this formulation has a clearer physiological reasoning than normalizing by the number of muscle elements or not introducing a normalization factor at all.

That the largest effect of the different muscle recruitment criteria was seen on the second peak of the knee contact forces is likely due to the relative change in muscle force estimates between gastrocnemius and soleus. In the TLEM dataset, soleus medialis and lateralis are each split into three branches, whereas gastrocnemius medialis and lateralis are each represented by one branch. As seen in the muscle force estimates, the different polynomial orders and normalization mostly affect the recruitment of these two muscles and as they are only active around the second peak and only gastrocnemius cross the knee, this is seen in the contact force estimates.

One of the underlying assumptions in the muscle recruitment formulation is that the muscles are activated solely to reduce the muscle activity to some power while satisfying the equilibrium equations and only pull. However, other conditions may also be at play, such as ensuring joint stability or neurological conditions that prevent optimal recruitment. In this case, increased co-contraction of the muscles will be expected, which will not be captured by this recruitment formulation (Forster et al. 2004). Such co-contraction will lead to an increase in the predicted knee contact forces. However, given the relatively close match to the total contact force for all three models applied in this study, it is not expected that a significant muscle co-contraction contribution has not been captured by the applied models.

Reduction of the isometric muscle strength of the knee flexors and extensors has been reported for TKA (Silva et al. 2003). As we have shown here, the predictions of the knee contact forces are highly sensitive to the specific strength of the knee flexors and extensors and, therefore, these should ideally be adjusted to the given patient. We unfortunately do not know whether the 35% reduction in strength of these muscles match this particular patient as isometric strength measurements were not available in the dataset. In this study, we chose to vary the knee flexor and extensor strength from 100% to 40% in steps of 10%. As we do not know the exact reduction range for TKA patients, we aimed to report the results for a range that likely include most TKA patients and that also included the case of 100% strength as that has been applied in multiple models of these patients (Fregly et al. 2012a,2012b). Additionally, as we found the effect of not accounting for the muscle strength reduction of TKA patients, i.e. 100% as compared to the 65% strength, had a larger effect on the predictions than switching between a linearly scaled and patient-specific model and inclusion of a detailed joint model, we encourage future studies of TKA patients to investigate the effects of including patient-specific strength measurements in the models.

Although speculative, assuming the muscle recruitment criterion is correct, a subject with normal or close to normal isometric strength of the knee flexors and extensors, but otherwise identical to this TKA subject, would have higher contact forces. Whether this is true is difficult to evaluate, as the forces can currently not be measured in an intact knee *in vivo*. In previous studies predicting knee contact forces (Fregly et al. 2012a,2012b), the muscle tendon parameters are rarely adjusted to the patients but rather based on cadaver data. With the sensitivity demonstrated here to the specific knee flexor and extensor strength, and knowledge of reduced knee strength for this patient group, we encourage future work to investigate this issue further. These results are in line with the previous study by Hast and Piazza (2013), who also found the predicted tibiofemoral forces to be sensitive to the specific muscle strengths and over-predicted the forces when the strengths were not reduced to account for the age-related reduction in strength.

Another uncertainty that musculoskeletal modelers are faced with in models using skin markers is the position of the markers on the model geometry when the only information available is trajectories of the skin marker in a laboratory coordinate system. In particular, the pelvis markers are problematic due to the amount of soft tissues surrounding the pelvic bone. As the specific distances from the bony landmarks to the actual skin marker positions, as well as the specific pelvic geometry are not known in these cases, the modeler must make a decision about where in between the markers the pelvic bone should be placed. Our results showed that moving pelvis anterior or posterior can have a large impact on the prediction of the second peak and moving pelvis a couple of centimeters can change the predictions by up to one body weight. We, therefore, suggest that specific experimental setups are developed to provide this important information for instance using ultrasound or through regression equations that relate to the size of the abdomen region.

Our results also showed that the inclusion of the patient-specific geometry and knee joint model did not improve the predictions compared to the linearly scaled model for this particular subject. For the total and medial knee contact force, the differences were minor whereas the linearly scaled model provided more accurate estimates of the lateral contact force. These results indicate that just because the model is made more anatomically accurate, it does not necessarily translate into improvements in the predictions of the knee contact forces. These results are in line with those of Correa et al. (2011) who found that, although muscle moment estimates were improved in their MRI-based models as compared to scaled generic models, this did not translate into a change in the predicted functional role of the muscles. However, our results are in disagreement with those of Gerus et al. (2013) who found improvements in their predictions when subject-specific geometry was applied. As our study was not designed to deduce the causes of why we did not see improvements in the contact forces, when patient-specific geometry was applied, we can only speculate about causes. First, the modeling approaches applied in our study differed from Gerus et al. (2013) in terms of model inputs and assumptions. Due to the nonlinearly of the musculoskeletal system, we cannot expect the same sensitivity of the model to the input geometry. Second, it is likely that, although the patient-specific scaling may have improved the muscle moment arms, the lack of patient-specific calibration of the muscle-tendon parameters, has placed the muscles on a non-ideal part of the force-length and force-velocity curves, which can counteract the effect of the moment arms. Further studies are required to clarify this though.

It has been demonstrated previously that linear and simple anisotropic scaling of geometric models can result in inaccurate moment arm and muscle tendon length estimates that both affect the force-generating capacity of the muscles (Scheys et al. 2008). However, as there are multiple other uncertainties in the models, improving the geometric representation may be outweighed by remaining uncertainties of the model, e.g. muscle recruitment criterion and muscle strengths.

The study has a couple of limitations that are worth discussing. Firstly, we only analyzed one subject during one gait trial and our results can, therefore, not be generalized beyond this subject. However, although only demonstrated on one subject, our results showed that including patient-specific geometry and a detailed joint model does not necessarily improve the predictions as our results provide a counterexample to that argument. Secondly, as the models were based on the TLEM 2.0 dataset, the results may not be generalizable to other datasets, e.g. TLEM 1.0 (Klein Horsman et al. 2007) or Delp et al. (1990). Thirdly, in the linearly scaled model, we applied a simple equilibrium in the frontal plane to distribute the total contact force and frontal moment to the medial and lateral side. As shown by Saliba et al. (2017), with such an approach, the estimated forces are sensitive to this distribution. However, the specific bone geometry and contact points are not available when applying generic models and only use skin marker trajectories and force plate data as input. Therefore, applying a regression model to relate the knee width to the moment arms of the medial and lateral contact force seems reasonable. Without going to full CT or magnetic resonance imaging (MRI)-based models, this can be improved by identifying the specific knee dimensions required for the equilibrium equations from a frontal X-ray. Fourthly, we only investigated a sub-set of possible muscle recruitment criteria and these results can, therefore, not be generalized beyond the specific criteria evaluated.

In conclusion, we found that the introduction of muscle normalization factors to account for sub-divided muscles affected the predicted knee contact forces mostly when a second-order muscle recruitment criterion was applied and the effect was primarily seen for the second peak. Among normalizing by the muscle volume or the number of muscle elements, normalizing by the number of muscle elements had the largest impact on the contact force predictions. The predictions were sensitive to the isometric strength of the knee flexor and extensors and the anterior–posterior position of the pelvis markers. Future studies should develop methods to reduce these uncertainties, such as personalizing the muscle-tendon parameters to isometric and isokinetic measurements, and improving the position of the pelvis markers relative to underlying bone, e.g. using ultrasound technologies.
